# Complete chemical and structural characterization of selenium-incorporated hydroxyapatite

**DOI:** 10.1007/s10856-021-06631-6

**Published:** 2021-12-23

**Authors:** Baris Alkan, Caner Durucan

**Affiliations:** 1 Department of Metallurgical and Materials Engineering, Middle East Technical University, 06800 Ankara, Turkey; 2 BIOMATEN Center of Excellence in Biomaterials and Tissue Engineering, Middle East Technical University, 06800 Ankara, Turkey

## Abstract

Hydroxyapatite (HAp) has long been used as synthetic bone tissue replacement material. Recent advances in this area have led to development of dual-functional bioceramics exhibiting high biocompability/osteoconductivity together with the therapeutic effect. Selenium, in that respect, is an effective therapeutic agent with promising antioxidant activity and anticancer effects. In this study, selenium-incorporated hydroxyapatite (HAp:Se) particles have been synthesized by modified aqueous precipitation method using calcium (Ca(NO_3_)_2_·4H_2_O) and phosphate ((NH_4_)_2_HPO_4_) salts and sodium selenite (Na_2_SeO_3_). The effects of selenium incorporation and post-synthesis calcination treatment (900–1100 °C) on physical, chemical properties and crystal structure of resultant HAp powders have been investigated. Complete chemical identification was performed with spectroscopical analyses including Fourier transform infrared and x-ray photoelectron spectroscopy to elucidate the mechanism and chemical nature of selenium incorporation in HAp. Meanwhile, detailed x-ray diffraction studies by Rietveld refinement have conducted to explain changes in the HAp crystal structure upon selenium incorporation.

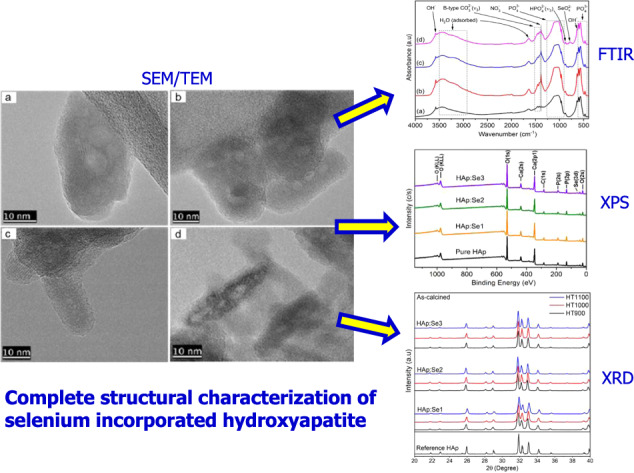

## Introduction

Calcium phosphates such as hydroxyapatite (Ca_10_(PO_4_)_6_(OH)_2_ or HAp) and calcium-deficient hydroxyapatite (Ca_(10−x)_(HPO_4_)_x_(PO_4_)_(6−x)_(OH)_(2−x)_ or CDHAp) have been widely used as hard tissue analogs due to their chemical similarity with natural bone mineral, as well as due to their superior bioactivity and osteoconductivity [1–6]. Mainly composed of calcium and phosphate the natural bone mineral is also a reservoir for various ionic species including sodium, magnesium, zinc, fluorine, carbonates etc. It is well known that different ion incorporations into HAp can alter cell regulation/signaling and eventually can change overall biological activity of HAp as regulated by specific interaction of these ionic components with cells and body fluids [7, 8]. Metallic ion-substituted systems with proper dopant amount may exhibit therapeutic action during osteointegration, yet not leading to any toxicity for surrounding bone cells. Particularly, copper and cobalt ions trigger blood vessel formation with induction of pro-angiogenic factor such as vascular endothelial growth factor (VEGF) while gallium and silver ions prevent bacterial infections and have been incorporated to bone tissue engineering scaffolds [9]. The ionic species of selenium, in this regard, are critically important since they constitute of selenoproteins which are essential to the immune system with their antioxidant function, and serve as catalyst in production of active thyroid hormone, and leading to cell proliferation [10].

In order to enable the expression of selenoproteins and protective roles of selenium species, selenium level in blood plasma should be at certain level. Several epidemiological studies have shown that selenium deficiency may amplify malignant tumor upturn in prostate, colon, lung, liver and throid pronouncedly [11, 12]. Also, low selenium intake can cause to increased levels of reactive oxygen species and phosphorylation that lead to inhibitory effect on collagen type-I, alkaline phosphatase expression and differentiation of osteoblasts that are regarded to act in expressing selenoproteins [13]. Thus, supranutritional selenium levels, higher than dietary supplementation are generally recommended in cancer treatments due to their chemo preventive effect against several cancer types including skin, breast, cervical ones as confirmed by the laboratory studies [14].

The potential anticancer effect of selenium is currently restricted to twelve different processes mainly regulated by the various selenium species [11]. Amongst these, L-selenomethionine, selenium-methyl L-selenocysteine, sodium selenite and sodium selenite are the most commonly used organic and inorganics forms of selenium. The functions of selenium species are complementary to each other in regard to selenoprotein expression against malignancy by inducing distinct cell apoptosis (death) mechanisms in terminating cancerous cells. Selenium-methyl L-selenocysteine is fundamentally effective against mutated cancerous cells that do not interact with p53 gene, while L-selenomethionine induce the apoptosis in specific cancerous cells that intact with this gene [15]. For sodium selenite, the apoptosis is induced on cancerous cells by diminishing a protein called Bcl-2 which is anomalously in high amount for cancerous cells [16, 17]. Overall, while being absorbed lower than organic selenium species, sodium selenite is more effective in inducing cancerous cell apoptosis due its higher genetic expression ability of glutathione peroxidase, the primary fundamental antioxidant enzyme containing selenium [18, 19]. Thus, sodium selenite can be potentially a promising and readily available selenium supplement in cancer treatment.

Different physical/chemical forms of selenium modifications have been implemented for orthopedic implant materials including elemental selenium coatings on titanium, use of nanostructured selenium compacts and selenite-substituted HAp powders [20–23]. The crystal structure related modifications upon selenium incorporation, which can be realized by different coating processes or during chemical synthesis of Se-incorporated powders appreciably affect the biological response of these systems in monoculture, co-culture cell studies and in vivo experiments as well [24]. Therefore, a refined analysis of structural properties of such modified systems is crucial. Motivated by this rationale, selenium-incorporated HAp (HAp:Se) particles have been synthesized by aqueous precipitation. Although there are several reports on determination of ion exchange mechanism in HAp and investigations on structural parameters in HAp lattice due to selenium substitution [25], there is not any through and in-depth chemical and structural analyses, elucidating the changes in HAp crystal structure upon Se incorporation. In this study, a complete chemical/structural refinement of HAp:Se has been performed elaborating with different complimentary analytical techniques and computational approaches. These findings may provide material-related insights in establishing the mechanism of selenium regulated biological/physiological response for Se-incorporated HAp, which can be achieved and somewhat controlled by varying the extent and crystal structure coordination/nature of selenium incorporation as shown in this current work.

## Experimental studies

### Materials and methods

Pure and selenium-incorporated HAp nanoparticles were synthesized using calcium nitrate tetrahydrate (Ca(NO_3_)_2_·4H_2_O, 99.0%; Sigma–Aldrich), ammonium hydrogenphosphate ((NH_4_)_2_HPO_4_, 98%; Sigma–Aldrich), sodium selenite (Na_2_SeO_3_, Bioreagent, suitable for cell culture, 98%; Sigma–Aldrich), ammonium hydroxide (NH_4_(OH), 26% NH_3_; Sigma–Aldrich) and ethanol (absolute, 99.8%; Sigma–Aldrich). All reagents were used without further purification. Ultrapure deionized (DI) water was used in all experiments.

0.25 M calcium nitrate tetrahydrate was first dissolved in 600 mL DI water at 25 °C. Meanwhile, the required theoretical amounts of ammonium hydrogenphosphate and sodium selenite were dissolved at 25 °C in separate beakers containing 600 mL DI water. Molar Se/P were adjusted as 1, 2, and 3% and ammonium hydroxide was then added to keep the solution pH at 10. Afterwards, calcium-containing solution was dropwise added into ammonium hydrogen phosphate-sodium selenite solutions using an automated injection pump (Top-5300 model syringe pump) at a rate of 60 mL/h in ambient conditions. The pH of the solution was monitored and ammonium hydroxide was constantly added during the injection process to keep solution with a pH at approximately 10. Once injection was completed, the parent solution was heated at 60 °C and aged at this temperature overnight.

Then, the precipitates were collected by centrifugation at 8000 rpm [using Eppendorf 5805 model (Eppendorf AG, Hamburg, Germany) for 5 min After that, the centrifuged particles were ultrasonically washed with DI water to remove nitrate residuals and also in ethanol solution for dispersion of agglomerated precipitates (for 30 min at room temperature using Branson 3510 model ultrasonic bath). Following ultrasonic treatments, the precipitates were collected by centrifugation at 10,000 rpm for 5 min and then dried on a borosilicate glass plate at 105 °C overnight. Afterwards, the dried solid precipitates were placed inside 250 mL high density polyethylene bottles (Nalgene^TM^) containing at least 50 mL of acetone (Acetone, absolute, ACS reagent 99.5%) and shaked by hand to wet the powders completely. Then, zirconia milling balls (10 mm in diameter, 30 of them) were placed inside the bottles, and milling was achieved using a 3D shaker mixer (Turbula, T2F Model, WAB, Switzerland) for 30 min.

Pure HAp was synthesized using the same experimental procedure by implementing a minor modification, where Ca/P ratio, controlled by calcium nitrate tetrahydrate and ammonium hydrogen phosphate aqueous solutions was adjusted as 1.67. Some of as-synthesized HAp and HAp:Se powders are calcined at 700–1100 °C and characterized afterwards. The ambient atmosphere calcinations were performed at a heating rate of 5 °C/min and 1 h soaking time. The fired samples cooled to room temperature in the furnace.

### Characterization

The morphology and size of HAp and HAp:Se powders were investigated using scanning electron microscopy (or SEM, Nova Nano 430-FEI). The samples were coated with conductive gold alloy prior to SEM investigations. The elemental composition all HAp powders were determined by energy dispersive x-ray spectroscopy (EDX) using JEOL 2100 F model SEM. The elemental analyses by EDX were carried out in at least eight different regions for each sample and the compositions are reported in average atomic percent. The oxygen was excluded as a compositional component in the computations due to its instability owing to low-atomic weight. In addition, ultrasonically dispersed representative powder samples have been examined using particle size analyzer (Malvern Mastersizer 2000). In typical experiment few droplet of nonionic surfactant (TWEEN 80) was added to the dispersed slurry and the powders were then exposed to ultrasonic treatment for 5 min before measurement.

The particle morphology of as-synthesized powders were also examined using JEOL JEM2100F model (JEOL Ltd., Tokyo, Japan) transmission electron microscope (TEM). TEM specimens were prepared by ultrasonic dispersion of agglomerated nanoparticles in ethanol solution for 10 min. Then, the TEM samples were obtained by depositing the powder suspensions on holey carbon-coated copper grids.

Fourier transform infrared analyses (FTIR, Frontier-PerkinElmer, equipped with PIKE GladiATR Reflection Specular Reflectance tools) was employed in order to reveal modifications in hydroxyl ion occupancy, incorporation of carbonate ions and specifically to identify selenium-related absorption bands, as well as other chemical structure alterations due to selenium incorporation. In preparation of FTIR samples pure and Se-incorporated HAp powders were mixed with KBr (Sigma, St. Louis, MO, USA; 95:5 wt. % of KBr:HAp) followed by pressing of the mixture to prepare the thin pellets.

The x-ray photoelectron spectroscopy (XPS) analyses were also performed for compositional identification, more specifically for determining the presence of carbonate ions and the chemical state of selenium for HAp:Se samples. The data acquisition was performed using PHI-5000 Versaprobe employing Al Kα radiation at 20 mA anode current, an electron accelerating voltage of 15 kV, a pass energy of 55 eV, and a step size of 0.1 eV. Both survey and regional high-resolution scans were acquired. The binding energies and charge corrections were referenced to the C(1 s) signal at 284.8 eV.

The crystallite size, microstrain, precise lattice constants and detailed structural parameters of HAp were calculated executing full profile refinement with both use of GSAS softwares [26]. XRD diffractograms obtained at a rate of 0.1 °/min were employed for profile and structural refinements. To obtain the instrumental parameters, standard reference material of LaB6 660b were used. Shifted Chebyschev function was used for background fitting depending on complexity of background patterns while peak shape function was selected as convoluted pseudo-Voight to consider anisotropic crystallite size and strain. In particular, as having a better fit than isotropic model, unaxial size and mustrain model was used to calculate equatorial and axial crystallite sizes. In the final stage of full pattern refinement, structural terms including atomic positions and site occupancies are simultaneously refined. Atomic displacement parameters (ADPs) were directly obtained from single crystal of Holly Springs HAp.

In order to determine the positions of Se- and C-related groups, difference Fourier maps (DELF) and peaks were used. For carbonate impurities in pure HAp structure, bond distances of C―O and O―O were soft restraint to the values of 1.294 and 2.241 and allowed to be refined. As to Se incorporated HAp, the atomic positions of incorporated ions were determined based on residual electron density from the corresponding DELF and satisfying bond length and angles. The obtained results were used to reveal nature of ionic incorporates, charge compensation mechanism and structural modifications within HAp lattice.

## Results and discussion

**Fig. 1:**
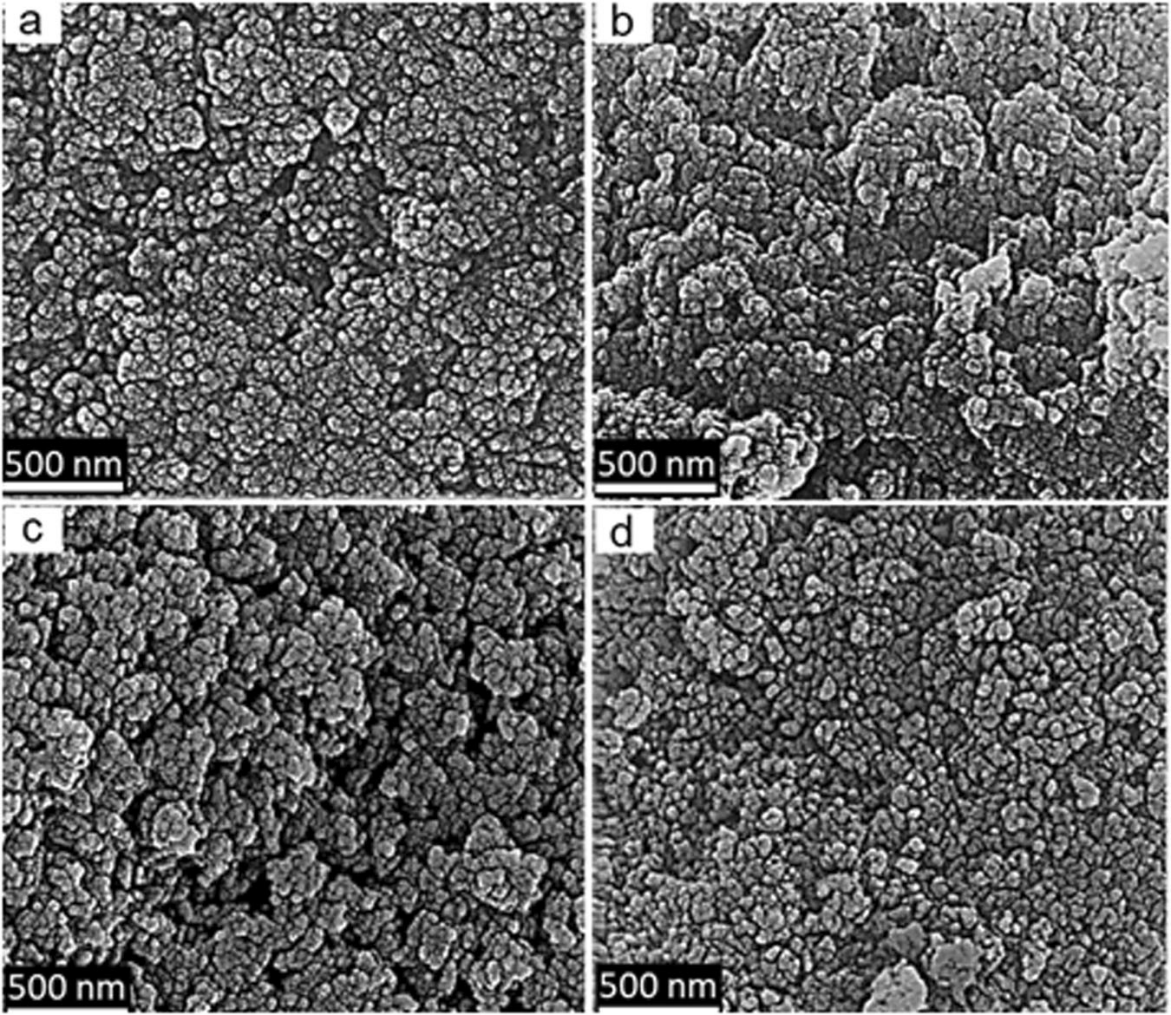
SEM micrographs of pure HAp (**a**), HAp:Se1 (**b**), HAp:Se2 (**c**) and HAp:Se3 (**d**) in as-synthesized condition

**Fig. 2:**
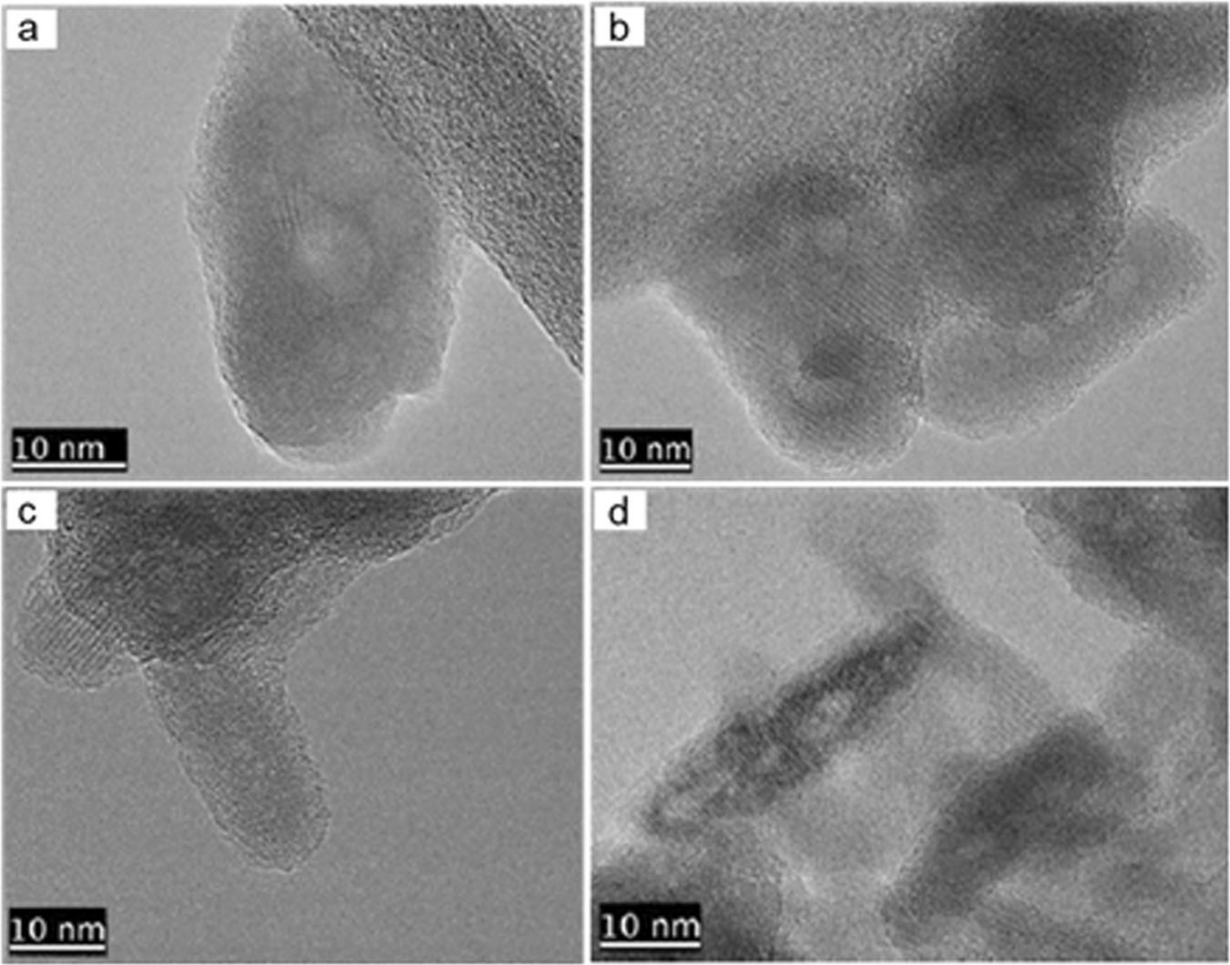
High-resolution TEM images of pure HAp (**a**), HAp:Se1 (**b**), HAp:Se2 (**c**) and HAp:Se3 (**d**) crystallites in as-synthesized condition

The SEM micrographs of pure and selenium-incorporated HAp nanoparticles are shown in Fig. 1. All particles are in a highly agglomerated state of submicron primary particles, having agglomerate size around 10 µm and show a monodisperse distribution within the agglomerates. Meanwhile, the morphological and size-related details of same nanoparticles are exhibited by the TEM images in Fig. 2. Apparently, all crystallites show anisotropic crystallite shape resembling a uniaxial (needle-like) morphology. Also, the crystallite anisotropy is observed to be more pronounced at higher Se-incorporation, suggesting a preferential growth for HAp crytal in axial directions upon Se incorporation

The chemical composition of pure and HAp:Se nanoparticles and atomic proportions as calculated by EDX analyses are shown in Table 1. Despite the considerable standard deviation from the computed mean values, the Ca/P and Ca/(P + Se) atomic ratios for pure and HAp:Se1 are observed to be slightly higher than those containing more selenium (HAp:Se2 and HAp:Se3). More importantly, Ca/(P + Se) atomic ratios were always calculated lower than the stoichiometric Ca/P ratio of 1.67 for HAp. This can be attributed to extensive deficiency of Ca^2+^ ions in HAp and HAp:Se structures to maintain charge neutrality disturbed by substitution of PO_4_^3−^ ions by SeO_x_^2−^ and/or CO_3_^2−^ ions. A previous study based on NMR analyses elaborated the variations in Ca/P ratio for HAp and indicated that the bulk and surface compositions and relevant ratios of constituent ions for HAp in nano-dimensions considerably changes with the respect to overall composition and ratio [27]. Hence, agglomeration state in fact affects the measured compositions and atomic ratios, as the core and the surface of HAp particles will be contributing to the calculated compositions with different extents. Nonetheless, the calculated Se/P percentages (in Table 1) were reasonably close to the expected ratios and imply that Se ions were well incorporated into HAp lattice. It is also worth to mention that, the Ca/P ratio of large agglomerates (greater than ~10 μm) is in the range of 1.58–1.63, which is more close to the stoichiometric Ca/P ratio of 1.67, while the Ca/P of smaller agglomerates (less than ~2 μm) was almost always close to 1.5. Size-dependent changes in Ca/P ratio can be interpreted that higher surface area in the smaller agglomerates directly increases the contribution surface composition to overall composition for HAp/HAp:Se particles thereby resulting in smaller Ca/P ratio.

**Table 1:** Bulk chemical compositions (in atomic %) of HAp and Se-incorporated HAps’ and elemental ratios as determined by EDX analyses

	HAp	HAp:Se1	HAp:Se2	HAp:Se3
Se/P % (nominal)	–	1.00	2.00	3.00
Se/P % (measured)	–	0.97 ± 0.05	1.79 ± 0.13	2.67 ± 0.27
Se (at. %)	–	0.39 ± 0.02	0.73 ± 0.06	0.99 ± 0.07
Ca (at. %)	59.24 ± 1.78	59.59 ± 1.24	58.78 ± 0.56	58.60 ± 1.00
P (at. %)	40.76 ± 1.78	40.02 ± 1.24	40.50 ± 0.52	40.41 ± 1.03
Ca/P, Ca/(P + Se)	1.45 ± 0.09	1.47 ± 0.08	1.43 ± 0.03	1.42 ± 0.02

**Fig. 3:**
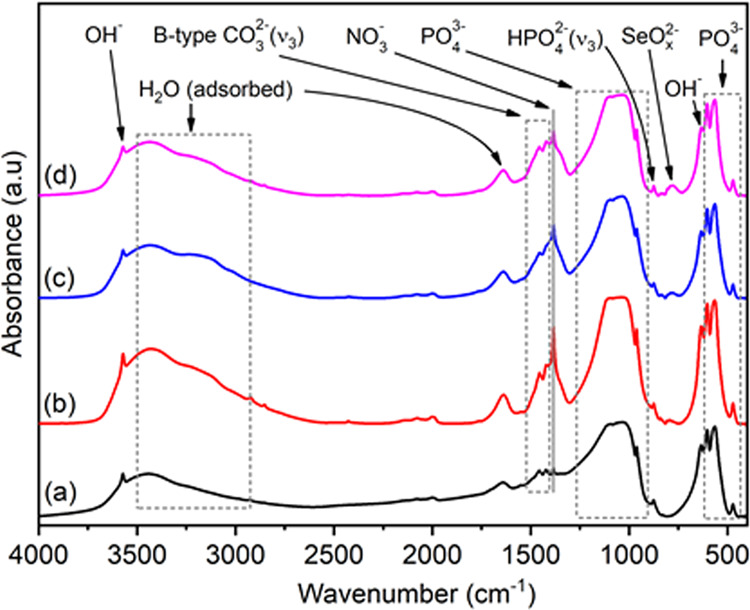
FTIR absorption spectra of pure HAp (**a**), HAp:Se1 (**b**), HAp:Se2 (**c**) and HAp:Se3 (**d**) in as-synthesized condition and the absorption bands of relevant chemical groups

The chemical structure of pure and Se-incorporated HAp powders were investigated using FTIR by relating the absorption events with the possible functional chemical groups of HAp. The general spectra (4000–400 cm^−1^) of pure and selenium-incorporated HAp samples together with the main ionic species and their corresponding absorption bands are indicated in Fig. 3. These spectra reveal the main absorption bands of phosphate groups (${{{{{\mathrm{PO}}}}}}_4^{3 - }$ ≈ 564, 575, 603, 961, 1031, 1092 cm^−1^) and hydroxyl group (${{{{{\mathrm{OH}}}}}}^ -$ ≈ 632, 3571 cm^−1^), and also the wide absorption band of physically adsorbed water in the range of 2600–3700 cm^−1^ as well as at 1640 cm^−1^ [28]. In addition, the bands at 1384 cm^−1^ was attributed to N – O stretching bands, more likely absorbed by KBr powders in the prepared pellets and the carbonate ions $({{{{{\mathrm{CO}}}}}}_3^{2 - })$ were detected in IR spectra in accordance with the absorption bands at around 1456, 1420 cm^−1^. These characteristic absorption bands are well-matched with B-type substitution of ${{{{{\mathrm{CO}}}}}}_3^{2 - }$ to ${{{{{\mathrm{PO}}}}}}_4^{3 - }$ sites [29, 30]. Considering the estimated formulation by Featherstone et al., relating the B-type carbonated content with the extinction coefficients of the bands near 1415 cm^−1^ of CO_3_ groups and 575 cm^−1^ of PO_4_ groups, the B-type carbonate contents for pure HAp, HAp:Se1, HAp:Se2 and HAp:Se3 were calculated as 3.99%, 3.26%, 5.68% and 4.86%, respectively [31]. These results suggest no direct correlation between carbonate and Se-related groups; however, the enhanced B-type carbonation was triggered with increasing Se oxyanion content.

**Fig. 4:**
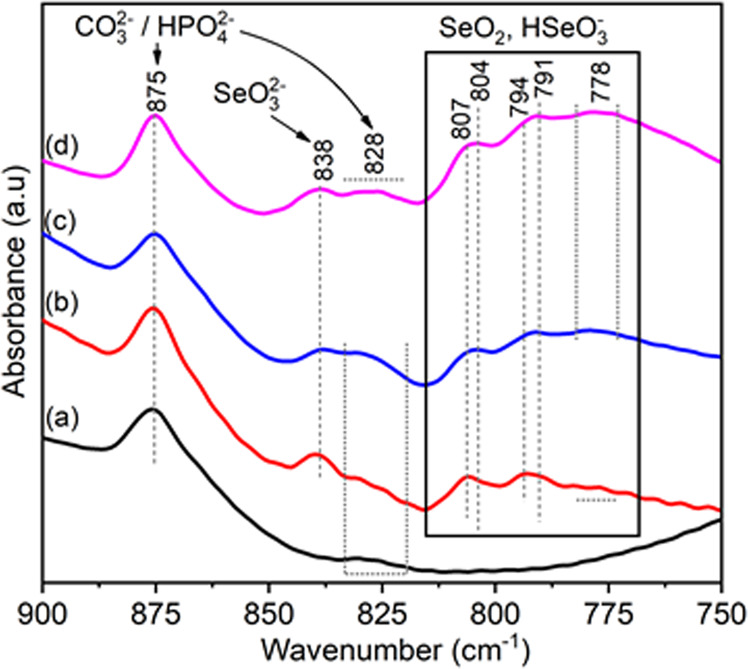
Regional (900–750 cm^−1^) FTIR spectra of pure HAp (**a**), HAp:Se1 (**b**), HAp:Se2 (**c**) and HAp:Se3 (**d**) in as-synthesized condition indicating the relevant Se oxyion groups as well as phosphate and carbonate groups at respective band positions

The detailed examination of 900–750 cm^−1^ spectral region, as shown by Fig. 4, indicates several differences for pure and Se-incorporated HAp samples. The band at 875 and the low intensity shoulder at 828 cm^−1^ are common and observed for all samples which are assigned to either CO_3_^2−^ or HPO_4_^2−^ groups [30]. The weak bands at 838, 791–794 and 778 cm^−1^ related to Se oxyanions are noticeable for Se-containing samples only and attributed to the asymmetric vibration of SeO_3_, SeO_2_ and SeO_2_ in HSeO_3_^−^, respectively [32]. Although not that distinctly appearing, the bands at 804–807 cm^−1^ are likely associated with SeO_2_ groups considering their band intensity profile exhibiting similarity to those positioned at 791–794 cm^−1^. These are also only observable for Se-incorporated HAp’ samples. One important observation in regard to band positioned at 838 cm^−1^ and set of absorption bands at 810–760 cm^−1^ (marked in the box in Fig. 4) indicating increasing content of SeO_2_ (selenite) groups at higher amount of Se incorporations.

**Fig. 5:**
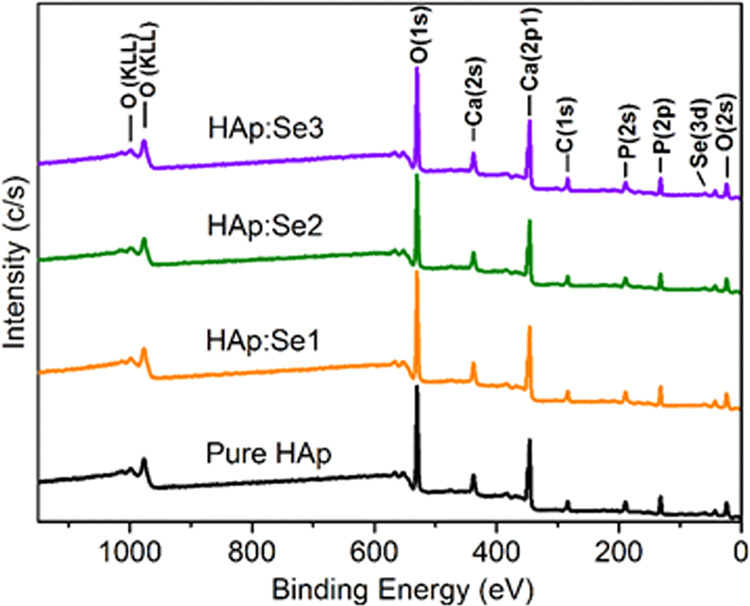
XPS spectra of pure and Se-incorporated HAp in as-synthesized condition

The chemical composition of pure and Se-substituted HAp powders, as well as the chemical state of Se-related species were also determined using XPS (through survey and corresponding regional scans) to further confirm the compositional findings obtained by EDX and FTIR analyses. The XPS survey spectra shown in Fig. 5 indicate the presence of all theoretically expected elements of HAp. Meanwhile, corresponding equivalent exact chemical compositions (excluding hydrogen) of all HAps are listed in Table 2. As expected, relative amount of P decreases gradually with increasing Se content. On the other hand, Ca amount (at around 17 at. %) and O amount (at around 54 at. %) is same for all samples. The total carbon amount is also observed to increase with increasing Se content, which can be more likely correlated with increasing content of CO_3_^2−^ (see Table 2). In addition, the calculated Se/P (at. %) are observed to be almost two times higher than the bulk Se/P (at. %) calculated by EDX (see Table 1), implying higher segregation of Se oxyanions in the crystallite surfaces. Also, Ca/(P + Se) at. ratios show a noticeable increase with higher Se substitutions while origin of this tendency is not clear due to coexistence of both C- and Se-groups. However, Ca/(P + Se) at. ratios vary in the range of 1.37–1.53 depending on the Se content and are comparable with the EDX-based values, which were found to at around 1.42–1.47 (see Table 1).

**Table 2:** The approximate chemical composition (in at. %) of pure and selenium-incorporated HAp powders computed based on XPS data

	HAp	HAp:Se1	HAp:Se2	HAp:Se3
P(2 s)	13.1	12.3	11.4	10.8
Se(3d)	–	0.3	0.4	0.6
Ca(2p)	17.8	17.3	17.6	17.4
O(1 s)	54.5	54.4	54.3	52.8
C(1 s)-total	14.7	15.7	16.3	18.4
C(1 s)-carbonate	1.6	2.5	2.9	3.6
Se/P (%)	–	2.4	3.5	5.5
Ca/(Se + P)	1.36	1.37	1.49	1.53

**Fig. 6:**
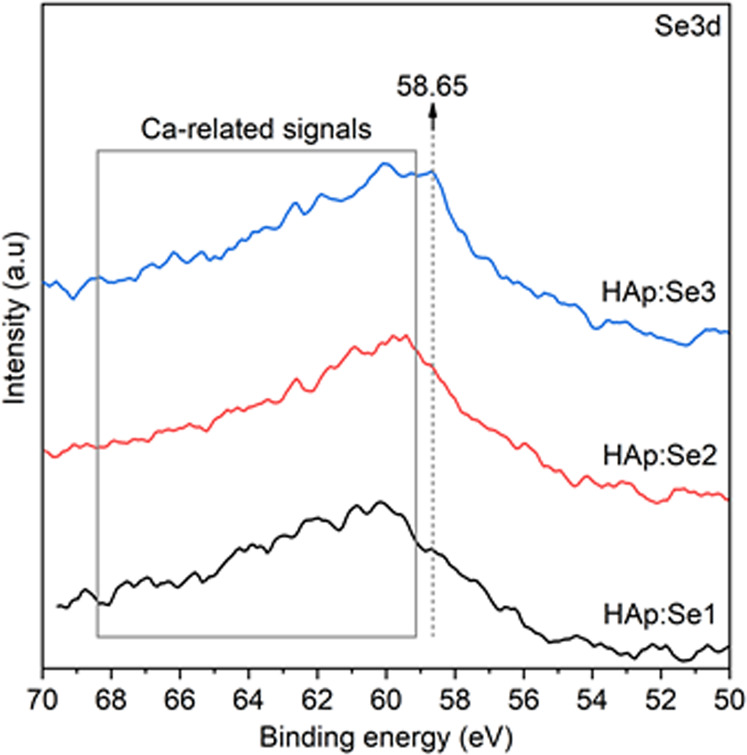
The regional XPS spectra of Se(3d) signal for Se-incorporated HAp powders in as-synthesized state

In order to clarify the chemical state of Se species, 70–50 eV region was examined for charge-corrected Se(3d) signal details (Fig. 6). The binding energies of metallic Se(3d_5/2_) and Se(3d_3/2_) are at around 54.6 eV and 55.5 eV, respectively [20]. A relatively higher (approx. 3–4 eV) binding energies for Se signal indicate that Se species are in ionic form and oxidized state It is worth to mention that, Se(3d) spectral region also includes some Ca-related signals just above the binding energies of Ca(3 s) between 60 and 65 eVs (marked with the boxed region) somewhat overlapping with the higher eV segment of Se(3d) signal [33]. These signals were attributed to the satellite loss features of Ca(3 s) analogous to the satellite loss features observed for Ca(2p) at 355 and 360 eV. Therefore, interpretation of Se(3d) signal is more precise for the binding energies lower than 59 eV. For precise quantification of Se and Ca some data refining was performed on the regional spectral data. The 70–50 eV spectral region signal was analyzed according the following protocol. First, the integrated intensity of the Ca1s satellite signal in 70–50 eV for pure HAp were determined and proportioned with the integrated intensity of the signature XPS signal of Ca(2p) signal at 350 eV, to obtain a fixed ratio between the signals of Ca1s satellite and Ca(2p). This proportion was then used to estimate the absolute intensity of Se(3d) signal in 70–50 eV region for Se-substituted HAp for quantification. In view of this remark, it was observed the intensity of 58.65 eV located signal amplifies with increasing Se content as shown in Fig. 6. This is assigned to Se^4+^ ions of SeO_3_^2−^ groups [20]. In addition, the relative contribution of Ca(1 s) satellite features are observed to be almost same for all HAp:Se’s excluding presence of Se^6+^ signal of ${{{{{\mathrm{SeO}}}}}}_4^{2 - }$(selenate), positioned at about 61 eV, which is expected as another possible Se-related chemical modification in the HAp [34]. As a whole, the findings based on the bulk analyses through FTIR and surface analyses through XPS spectra suggest that Se-substitution occurs in the form of SeO_3_^2−^; therefore, highlighting the existence of bulk and surface Se^4+^ ions in both Se-substituted HAp.

**Fig. 7:**
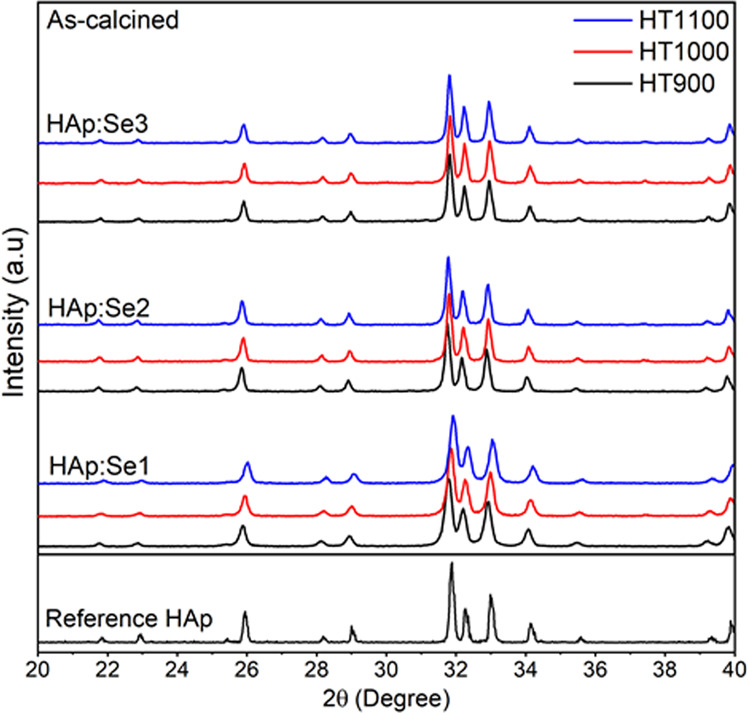
Diffractograms of pure and selenium-incorporated HAp powders at as-synthesized, condition and after calcination at 900 °C, 1000 °C and 1100 °C, representing high temperature stability of these powders. The reference HAp (pattern for JCPDS 9-432) is shown for comparison purposes

**Fig. 8:**
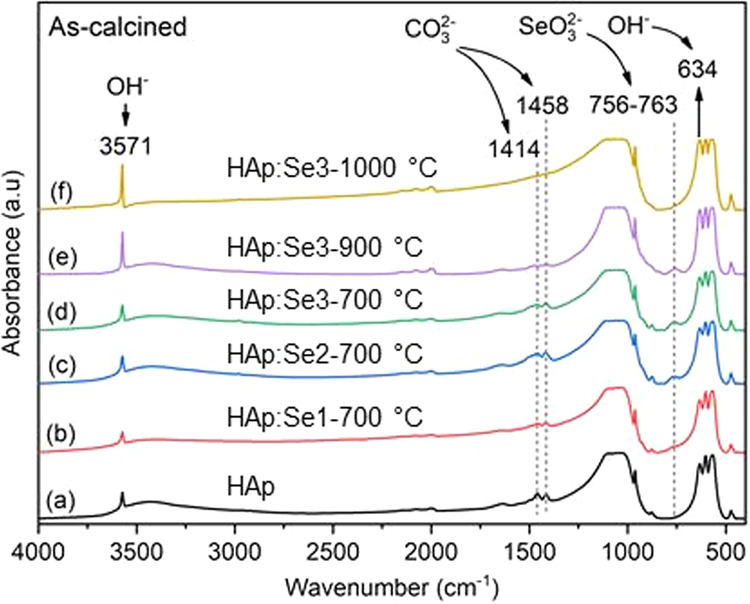
FTIR spectra of pure HAp (**a**), HAp:Se1 (**b**), HAp:Se2 (**c**) and HAp:Se3 (**d**) calcined at 700 °C. Additional FTIR spectra of as-calcined HAp:Se3 at 900 °C (**e**) and at 1000 °C (**f**) are shown to exhibit the effect of high temperature calcination on thermal stability of the incorporated functional groups

High temperature stability of HAp:Se powders were investigated using XRD and FTIR. The diffractograms of the powders fired at 900°, 1000° and 1100 °C are presented in Fig. 7. Again, there was not secondary phase formation in any case. However, FTIR spectra (in Fig. 8) show the effects of the calcination temperature on the functional groups constituting the HAp. One clear distinct change is the intensity of OH^−^ bands at 634 and 3571 cm^−1^, which can be more easily depicted from the data for HAp:Se3. The 634 cm^−1^ band intensity increases gradually comparing the spectra of 700° and 1000 °C-calcined samples (labeled as d–e in Fig. 8). This is also valid for the other OH^−^ band located at 3571 cm^−1^ and its’ intensity increases with increasing calcination temperature. Meanwhile, the intensity absorption bands at 1414 and 1458 cm^−1^ assigned for B-type CO_3_^2−^ ion decreases upon heating from 700 °C to 900 °C. In addition the absorption band for selenite (756–763 cm^−1^) completely disappears for 1000 °C-calcined HAp:Se3. These all together show that after calcinations at temperature higher than 1000 °C, both the B-type carbonate ions and selenite ions are completely removed from HAp. This implies that removal of Se substitutes from HAp lattice may be possible during sintering of HAp:Se or during other high-temperature processes involving use of these powders. Therefore, firing temperature can be 900 °C at maximum to keep Se oxyanions within HAp structure.

**Fig. 9:**
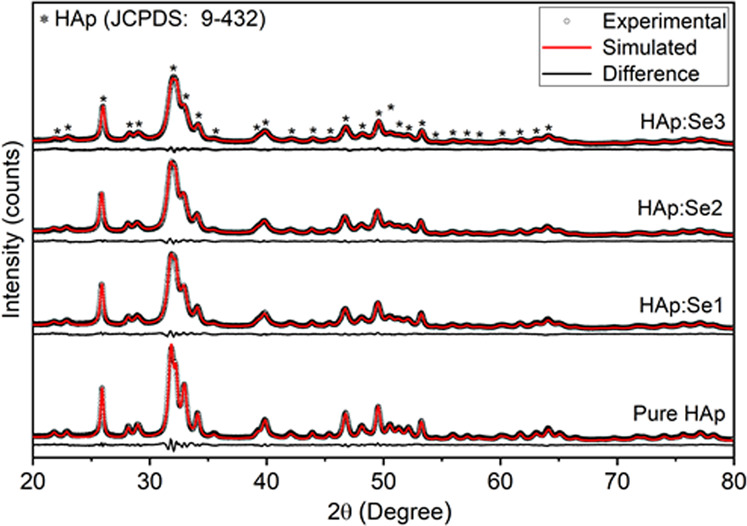
XRD diffractograms of pure and Se-incorporated HAp samples as-synthesized condition. Note that all patterns show only the diffraction peaks of HAp with JCPDS no. of 9-432

The structural modifications and Se-incorporation mechanism/nature in HAp lattice as function of Se content was studied by XRD analyses and by full Rietveld refinement through GSAS software [26] for as-synthesized powders. Figure 9 demonstrates the slow-scanned diffractograms of pure and Se-incorporated HAp powders, as well as the result of the refined powder patterns through GSAS software. Qualitative phase analyses reveal that there was not any secondary phase formation in all cases. Compared to those of pure HAp, relatively lower peak intensities for Se-incorporated samples and the distortion of well-defined diffraction peak shapes for HAp:Se patterns suggest that crystallinity may be diminished upon Se incorporation. Also, broadening of the diffraction peaks with Se incorporation can be attributed to a reduction in crystallite size. and/or an increase in microstrain with increasing Se content.

To support the qualitative information obtained from XRD analyses, full profile refinements using carbonated HAp model (space group: P6_3_/m) were carried out and the refinement plots were shown in Both experimental and simulated plots of all XRD patterns were consistent with one another as it was confirmed by both accurate profile fitting in their corresponding plots and the reduced chi-square and R_wd_ values are given in Table 3. Accordingly, the calculated lattice parameters of HAp were observed to increase systematically with extent of Se incorporation. This tendency in the lattice parameters can likely be relevant to increasing microstrains in HAp:Se crystallites since the calculated microstrains enhance noticeably, in the equatorial direction of crystallites with increasing Se contents. In addition, the crystallite sizes, calculated using uniaxial crystallite size model, are observed to reduce both in equatorial and axial direction at higher Se contents. Thus, both reducing crystallite size and increasing microstrain can be reasoned for increased peak broadening at high Se contents.

**Table 3:** The refined peak profile and unit cell parameters as well as reduced chi-square values of the final refinements obtained from XRD diffractograms of as-synthesized samples

		HAp	HAp:Se1	HAp:Se2	HAp:Se3
χ^2^		1.38	1.13	1.24	1.28
R_wd_ (%)		4.01	3.29	3.35	3.41
Crystallite Size (nm)	Equatorial	22.4 (1)	16.6 (2)	16.2 (1)	14.4 (1)
	Axial	45.8 (5)	39.0 (6)	34.8 (4)	31.9 (3)
Mustrain (µstrain)	Equtorial	2.8 (1)e3	4.4 (4)e3	5.8 (3)e3	6.5 (3)e3
	Axial	1.3 (1)e3	1.9 (2)e3	1.9 (1)e3	2.0 (1)e3
Lattice parameter (Å)	a	9.4237 (2)	9.4273 (3)	9.4291 (3)	9.4339 (3)
	c	6.8839 (1)	6.8823 (2)	6.8855 (2)	6.8863 (2)

Before the refinement of structural parameters, substituted ions were included in structural models of HAp and HAp:Se3 (highest amount of Se incorporation). In this regard, for locating the CO_3_^2−^ ions around PO_4_^3−^ tetrahedra for pure HAp, the well-established models of Wilson et al. [35] and Fleet et al. [36] on B-type carbonated HAp structures were employed. Following the bases of these models, total composition of phosphate and carbonate ions were constrained to 6. Also, O−O bond distances in carbonate structure is restrained to 2.241 Å with a tolerance of 0.02 Å. The oxygen ions involved in the structural model of HAp were denoted by Oa, Ob and Oc, respectively.

To determine the positions of Se-related and CO_3_^2−^ ions in HAp:Se3, the residual electron scatterings and difference Fourier maps were investigated. First, high residual scatterings around PO_4_^3−^ were found and the some Fourier peaks of these residual scattering were calculated as 2.1530(1)Å, 2.2375(1)Å and 2.2893(1)Å and the corresponding bond angles are calculated as 60.39(0)°, 62.82(0)° and 56.78(0)°, respectively. The resultant bond lengths and angles are reasonable with the O−O bond distances and angles for CO_3_^2−^ ions in the previous studies (referring to calcite structure). Accordingly, the found residual peak (denoted by O1-C) was involved in the positional parameters of HAp:Se3. Second, residing near OH^−^ channel, additional residual electron scatterings (denoted by O2-C) were also found to be related to the oxygen ions in another CO_3_^2−^ groups. In particular, the bond distances between the Fourier peaks of the residual scattering and the mirror symmetric oxygen ions (O3 and O3′) in PO_4_^3−^ tetrahedra were computed as 2.2476(1)Å while the distance between the symmetric oxygen atoms were computed as 2.4478(1)Å. The found peak distances together with the corresponing bond angles about 60° suggest the presence of CO_3_^2−^ ions near OH^−^ in the structural model of HAp:Se3.

The Fourier peaks of the residual scatterings obeying the structural features of SeO_3_^2−^ and SeO_4_^2−^ were also investigated in DELF maps of HAp:Se3. No Se-related residual scattering could be found close to the PO_4_^3−^ tetraheda. However, the residual scatterings situated in the octahedral holes between Ca (I) ions were found to be relevant to Se-related ions as the positional differences between these residual scatterings were calculated 1.7037(1)Å and 96.26(0)°, respectively. These results almost correlate with the average bond length (Se−O) and angles (O−Se−O) of SeO_3_^2−^ ions in the previous reports [37, 38]. Thus, the atomic positions of Se were also involved in the structural model for HAp:Se3.

Following the additional atomic positions found in DELF maps, the refinement of the atomic positions and occupancies of pure HAp and HAp:Se3 are carried out and their result are tabulated in Table 4. The calculated site occupancies of oxygen ions in CO_3_^2−^ and OH^−^ groups as well as those of Ca^2+^ ions were observed to reduce in both samples. However, the site occupancy of Ca^2+^ ions are observed to be considerably low in HAp:Se3 compared to those of HAp, which can be more likely due to the charge compensation of the extra positive charge owing to the SeO_3_^2−^ ions as well as lowered occupancies of PO_4_^3−^ ions [39].

**Table 4:** The refined atomic positions and occupancies of the as-synthesized XRD patterns of pure HAp and HAp:Se3

	Equi-point	F	x	y	z	U_iso_
HAp						
Ca(I)	4 f	0.935	0.3333	0.6667	0.0013 (4)	0.0053
Ca(II)	6 h	0.926	0.2452 (2)	0.9896 (3)	0.2500	0.0048
P	6 h	0.896	0.3989 (4)	0.3696 (4)	0.2500	0.0030
O1	6 h	0.896	0.3319 (5)	0.4898 (5)	0.2500	0.0066
O2	6 h	0.896	0.5885 (6)	0.4682 (4)	0.2500	0.0106
O3	12i	0.896	0.3452 (4)	0.2601 (6)	0.0713 (8)	0.0124
O-OH	4e	0.439	0	0	0.186 (1)	0.0133
H	4e	0.500	0	0	0.0608	0.025
Oa	6 h	0.104	0.3303 (5)	0.4850 (5)	0.25	0.012
Ob	6 h	0.104	0.5561 (6)	0.4410 (6)	0.25	0.012
Oc	12i	0.052	0.3266 (6)	0.2713 (8)	0.0944 (7)	0.012
HAp:Se3						
Ca(I)	4 f	0.791 (2)	0.3333	0.6667	0.0013 (6)	0.0053
Ca(II)	6 h	0.789 (2)	0.2410 (2)	0.9847 (3)	0.2500	0.0048
P	6 h	0.781 (1)	0.3994 (4)	0.3683 (3)	0.2500	0.0030
O1	6 h	0.825 (2)	0.3308 (7)	0.4929 (7)	0.2500	0.0066
O2	6 h	0.825 (2)	0.5852 (8)	0.4632 (8)	0.2500	0.0106
O3	12i	0.825 (2)	0.3470 (4)	0.2611 (5)	0.0754 (4)	0.0124
O-OH	4e	0.451 (2)	0	0	0.181 (1)	0.0133
H	4e	0.500	0	0	0.0608	0.025
O1-C	6 h	0.061 (6)	03884 (7)	0.3530 (7)	0.75	0.012
O2-C	6 h	0.044 (5)	0.1193 (5)	0.1201 (5)	0.25	0.012
Se	4 f	0.014	0.3333	0.6667	0.8763	0.003

The weight percent of B-type carbonate ions through the refined site occupancies of the oxygen ions in CO_3_^2−^ structure were calculated as 4% and 4.5% for HAp and HAp:Se3, respectively. These results were compared to the estimated carbonate contents based on the formula described by LeGeros [40]. Accordingly, the carbonate contents resulted in 2.9% and 4.25 wt.% carbonate ions for HAp and HAp:Se3, respectively. Noticeably, the CO_3_^2−^ contents in HAp:Se3 are well in agreement with those based on theoretical formula (4.5% vs. 4.25%). For pure HAp, the slight differences between the CO_3_^2−^ (4% vs 2.9%) suggest further optimization of the structural model of HAp.

Based on the site occupancies calculated, the atomic ratios of Ca/P and Ca/(P + Se) for HAp and HAp:Se3 were obtained as 1.73 and 1.66, respectively. However, taking the extent of the incorporated CO_3_^2−^ within pure HAp and HAp:Se3 into account, the calculated ratios correspond to 1.55 and 1.46, lower values that are comparable with the atomic ratios obtained by the bulk chemical analyses by EDX. Also, atomic percent of Se/P ratio was obtained close to 1.45 which is roughly half of the bulk Se/P of 2.67. This result can be considered to be reasonable in that high Se/P atomic percent found in surface composition can result in the lowered Se content in the HAp:Se3 crystal composition. However, it is also noteworthy to consider the possibility of insufficient modeling of HAp:Se3 structure; therefore, requiring advanced crystallographic techniques to confirm the model proposed here.

## Conclusions

Selenium-incorporated submicron-size hydroxyapatite (HAp) were synthesized by modifying the conventional aqueous precipitation route used for obtaining phase pure HAp, by employing selenium salts during precipitation. It was found that, selenium substitution leads to selenite-based incorporates within HAp lattice. Also, selenium incorporation leads to calcium-deficient B-type carbonated HAp, which may intrinsically enhance osteoinductive properties of HAp due its higher chemical resemblance with natural bone tissue mineral, which is a calcium-deficient carbonated HAp as well. The structural refinements revealed presence of B-type carbonate ions nearby phosphate tetrahedral, whereas the existence of selenite-based ions within octahedral holes between Ca ions in HAp lattice. These findings can be useful in relating the structural details of Se- and carbonate-substituted HAp with their ionic release behavior in physiological environment governing their bioactivity and therapeutic function. With establishment of such relationship, ion-incorporated HAp can be possibly utilized in cancerogenic bone defect filling operations or they can be combined with polymeric or inorganic natural/synthetic bone analogs to impart therapeutic/regenerative functions to these systems.
